# The clinicopathological features and treatment modalities associated with survival of neuroendocrine cervical carcinoma in a Chinese population

**DOI:** 10.1186/s12885-018-5147-2

**Published:** 2019-01-07

**Authors:** Xiaojing Zhang, Zunfu Lv, Hanmei Lou

**Affiliations:** 1 Department of Gynecological Oncology, Zhejiang Cancer Hospital, 1 Banshan East Road, Hangzhou, 310022 People’s Republic of China; 2 Department of Agriculture and Food Science, Zhejiang A&F University, Lin’an, 311300 People’s Republic of China

**Keywords:** Neuroendocrine cervical carcinoma (NECC), Survival, NACT, RT

## Abstract

**Background:**

Neuroendocrine cervical carcinoma (NECC) is a rare but aggressive form of cervical cancer representing less than 3% of all cervical cancer cases. The objective of this study is to evaluate the effects of the clinicopathologic features and treatment modalities on the survival of patients with NECC.

**Methods:**

In all, 89 stage I-IV patients with NECC that were diagnosed and treated between 2006 and 2014 at the Zhejiang Cancer Hospital were retrospectively recruited in this study. The Kaplan-Meier method, Cox regression analysis models and the log-rank test were used for the statistical analyses.

**Results:**

NECC patients with advanced FIGO stage, tumor size > 4 cm, lymph node metastasis (LNM) and lymph-vascular space invasion (LVSI) were more likely to have significantly worse survival. Neither neo-adjuvant chemotherapy (NACT) nor radiotherapy (RT) was associated with improved overall survival. In the stratified analysis of stage I-IIA patients, those with advanced FIGO stage (*P* = 0.018), LNM (*P* = 0.008) and LVSI (*P* = 0.024) were associated with significantly worse survival. Patients without LNM who did not receive RT had significantly better survival rates than those who received RT (HR = 3.363, 95%CI = 1.245–10.619; *P* = 0.018). Moreover, for stage I-IIA patients with tumor size > 4 cm, NACT was not associated with a significantly better survival rate compared with no NACT (*P* = 0.600). None of the clinicopathologic features or treatment modalities was an independent prognostic factor in the multivariate analysis.

**Conclusions:**

In conclusion, advanced FIGO stage, tumor size > 4 cm, LNM and LVSI were associated with poor survival. For stage I-IIA patients, RT should be carefully used in patients who are negative for LNM, and NACT may not be the optimal treatment for patients with tumor size > 4 cm.

## Background

Neuroendocrine cervical carcinoma (NECC) is a rare but aggressive form of cervical cancer, as it accounts for less than 3% of all cervical cancer cases [1].

NECC consists of four histologic subtypes (small cell neuroendocrine carcinoma, large cell neuroendocrine carcinoma, typical carcinoid tumor, and atypical carcinoid tumor) [2]. The most common type of NECC is small cell carcinoma of the cervix (SCCC), which seems to be highly aggressive and to have a worse prognosis compared with squamous cell carcinoma and adenocarcinoma of the uterine cervix [3, 4]. Although NECC can also be mixed with other histologic types, such as squamous cell and adenocarcinoma, and the presence of the NECC component defines the clinical behaviour, the tumor should be treated according to the therapeutic regimen of NECC [2].

As we know, primary surgery and primary radiation therapy result in similar survival rates for early-stage cervical cancer of common histological types [5]. However, for NECC tumors that are ≤4 cm in size, the Society of Gynaecologic Oncology (SGO) and the Gynaecologic Cancer InterGroup (GCIG) both recommend that radical hysterectomy with lymph adenectomy followed by the administration of adjuvant etoposide/platinum-based therapies should be the preferred choice of treatment [2, 3]. For NECC tumors that are > 4 cm in size, neo-adjuvant chemotherapy (NACT) is recommended, followed by comprehensive treatment including surgery, once the tumor has been limited. These recommendations are based on limited data and are instructive for the basic treatment of NECC, but the complex treatment of NECC is not uniform in many places.

The aim of the present study is to evaluate the prognostic factors of overall survival and to examine the effects of treatment on the overall survival of patients with NECC.

## Methods

### Study populations

This study included a total of 93 patients who were diagnosed with NECC and who were treated at Zhejiang Cancer Hospital from January 2006 to December 2014. Overall survival (defined as the duration from primary treatment to death or the last follow-up) was the main study endpoint. Within the maximum 100-month follow-up period, four patients were excluded from our study due to a lack of sufficient follow-up information.

### Collection of outcome data

Information regarding the patient characteristics, treatment methods and results, site of recurrence, overall survival, and final outcome was collected in the study. Clinicopathologic data including age, FIGO stage, tumor size, histologic type, lymph node metastasis (LNM), lymph–vascular space invasion (LVSI), depth of stromal invasion (DOI), and distant metastasis status were obtained by reviewing their medical records. Two of the authors independently extracted the data. Disagreements were solved by consensus. All follow-up data were collected through inpatient and outpatient records and by telephone follow-up.

### Statistical analysis

The survival rates were calculated and estimated by the Kaplan–Meier method and were compared using the log-rank test. Univariate and multivariate Cox regression analyses were used to analyse the prognostic factors for overall survival. All *P* values in this study were 2-sided. *P* < 0.05 was considered statistically significant. All data were analysed using SPSS statistical software package.

## Results

### Patient characteristics and treatment

In all, data for 93 patients were retrieved, but four patients lacked survival data. The characteristics and clinicopathological features of the 89 eligible patients diagnosed with NECC are summarized in Table 1. The median age at diagnosis was 40.5 years (range 16–70 years), and the median overall survival of all the patients was 57 months. Among the patients treated by radical hysterectomy, LNM and LVSI were found in 23 cases (31.5%) and 27cases (37%), respectively. The obturator nodes were positive in 20 out of 23 patients. Nine patients had LNM to the iliac vessels. The distribution of the FIGO clinical stage among these patients was as follows: FIGO IA, 2 (2.2%); FIGO IB1, 34 (38.2%); IB2, 17 (19.1%); IIA1, 9 (10.1%); IIA2, 14 (15.7%); IIB, 5 (5.6%); III, 0; IVB, 8 (8.9%).

**Table 1:** Stage I-IV patients’ characteristics and clinical features

Parameters	Group (no.)	MST (mo)	HR	*P*
FIGO	I-IIA (76)	60		
	IIB-IV(13)	30	2.629(1.201–5.727)	0.016
Size	⩽4 cm (47)	65		
	>4 cm (42)	49	2.027(1.087–3.780)	0.026
LNM	No (50)	68		
	Yes(23)	44	2.573(1.269–5.216)	0.009
LVSI	No (46)	69		
	Yes(27)	46	2.443 (1.197–4.986)	0.014
DOI	⩽2/3 (57)	67		
	>2/3(16)	51	2.165(0.931–5.035)	0.073
NACT	No (35)	60		
	Yes(38)	59	1.166(0.567–2.401)	0.676
Radiotherapy	No (66)	61		
	Yes(23)	37	1.345 (0.676–2.678)	0.398
Syn	No (8)	36		
	Yes(39)	58	1.671 (0.383–7.280)	0.494
CD56	No (12)	77		
	Yes(30)	52	1.844 (0.606–5.612)	0.281
CgA	No (15)	76		
	Yes(31)	50	1.933 (0.635–5.882)	0.246
NSE	No (6)	44		
	Yes (10)	55	0.837 (0.197–3.549)	0.809

The treatment regimen for patients that were initially treated with primary surgical treatment included cancer-directed surgery (CDS) alone, CDS with adjuvant treatment (chemotherapy with or without radiotherapy) and surgery preceded by NACT with subsequent adjuvant treatment. Primary radiation therapy was concurrent chemoradiation. Fourteen patients received primary chemotherapy alone.

The numbers of patients that underwent surgery as their primary treatment were as follows: FIGO IA, 1; FIGO IB1, 31; IB2, 16; IIA1, 7; IIA2, 13; IIB, 4; III, 0 (13%); IV, 2; NACT was administered to 10 patients with stage IB1, 8 with stage IB2, 6 with stage IIA1, 9 with stage IIA2, 3 with stage IIB, and 2 with stage IVB disease. All 73 patients who underwent surgery also received postoperative adjuvant chemotherapy (PACT) or chemoradiotherapy. The most frequently used chemotherapy regimen was EP (etoposide and cisplatin), which consisted of 2–3 cycles for NACT, 3-4 cycles for PACT, or 6 cycles for chemotherapy alone.

### Effects of the clinicopathological features and treatment modalities on NECC survival

**Fig. 1:**
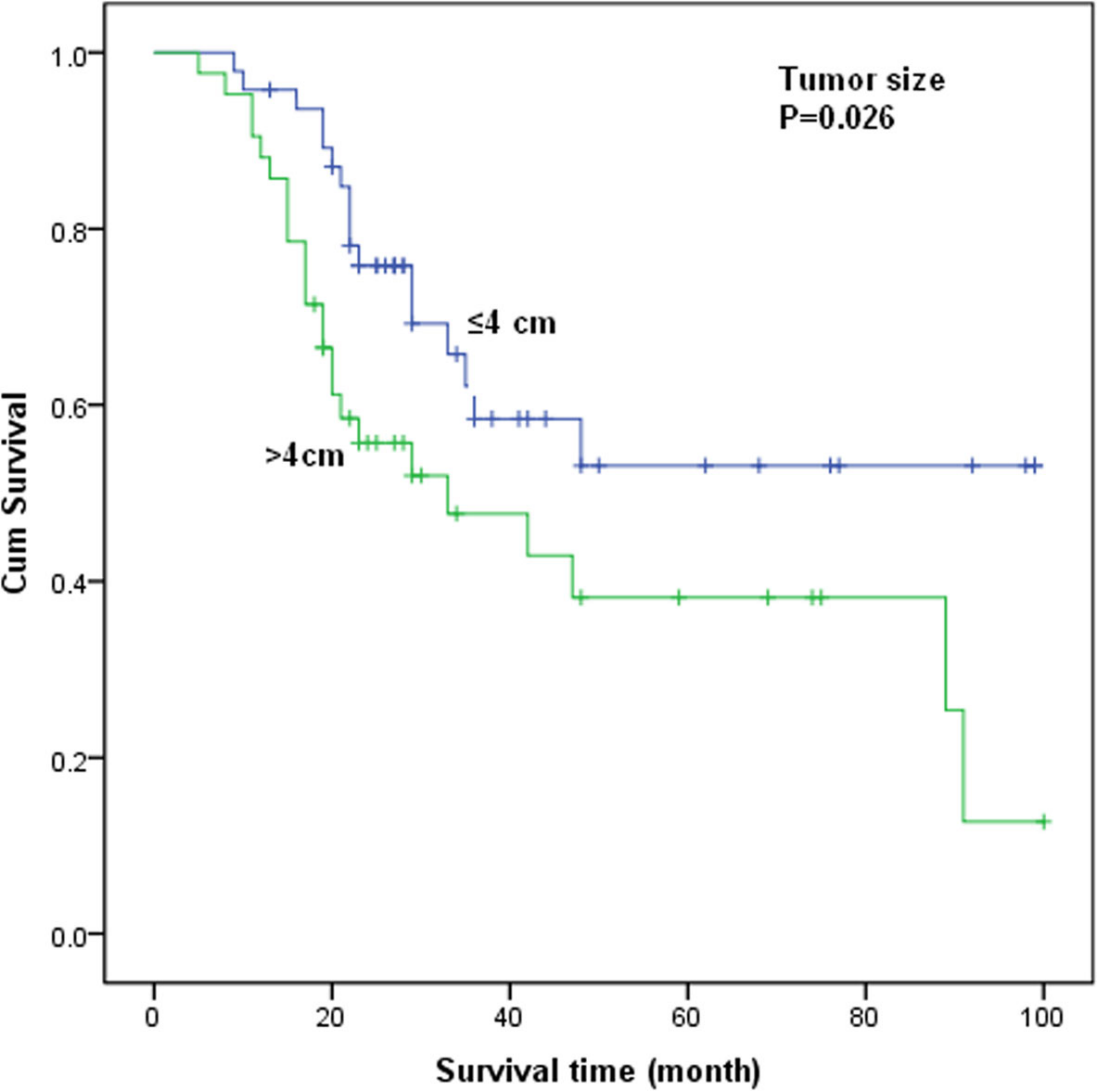
Kaplan-Meiercurves of overall survival for stage I-IV patients with NECC by tumor size

In the univariate analysis, compared with the early-stage patients (≤IIA), the median survival time (MST) of advanced–stage patients (≥IIB) with NECC decreased accordingly (*P* = 0.016). As for tumor size, there was a significant difference in overall survival time between tumor size > 4 cm and ≤ 4 cm (*P* = 0.026, Fig. 1). Patients with LNM (*P* = 0.009) and LVSI (*P* = 0.014) were associated with a significantly worse survival. However, as for the treatment modalities, neither NACT (HR = 1.166, 95%CI = 0.567–2.401) nor RT (HR = 1.345, 95%CI = 0.676–2.678) showed significant difference in the survival of patients with NECC. By immunohistochemistry, three neuroendocrine markers (synaptophysin, chromogranin, and CD56) showed a similar frequency of staining. Syn, CD56, CgA and NSE were not identified as prognostic factors for the survival of patients with NECC.

### Stratified analyses among stage I-IIA NECC

**Fig. 2:**
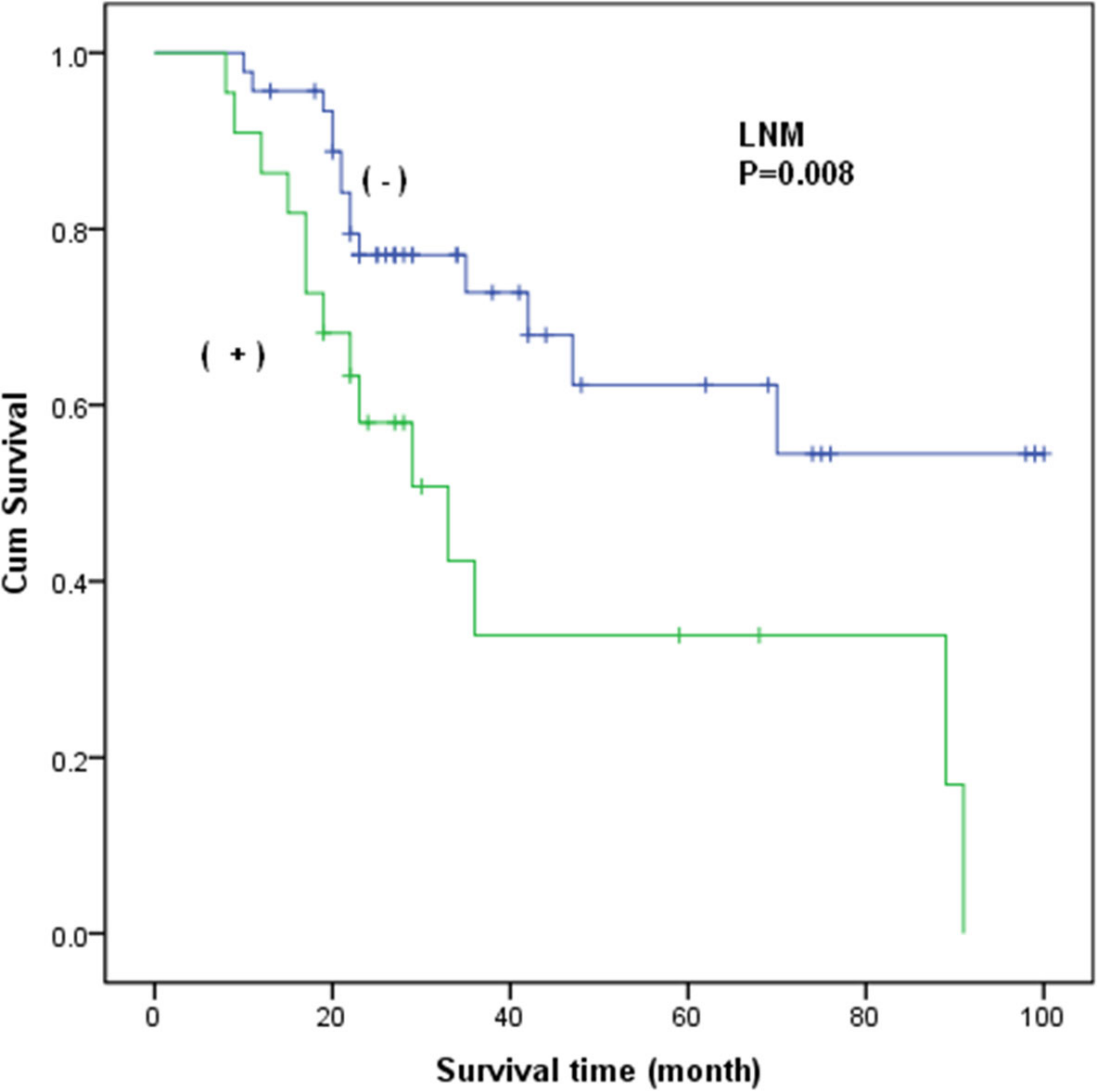
Kaplan-Meiercurves of overall survival for stage I-IIA patients with NECC by LNM

**Fig. 3:**
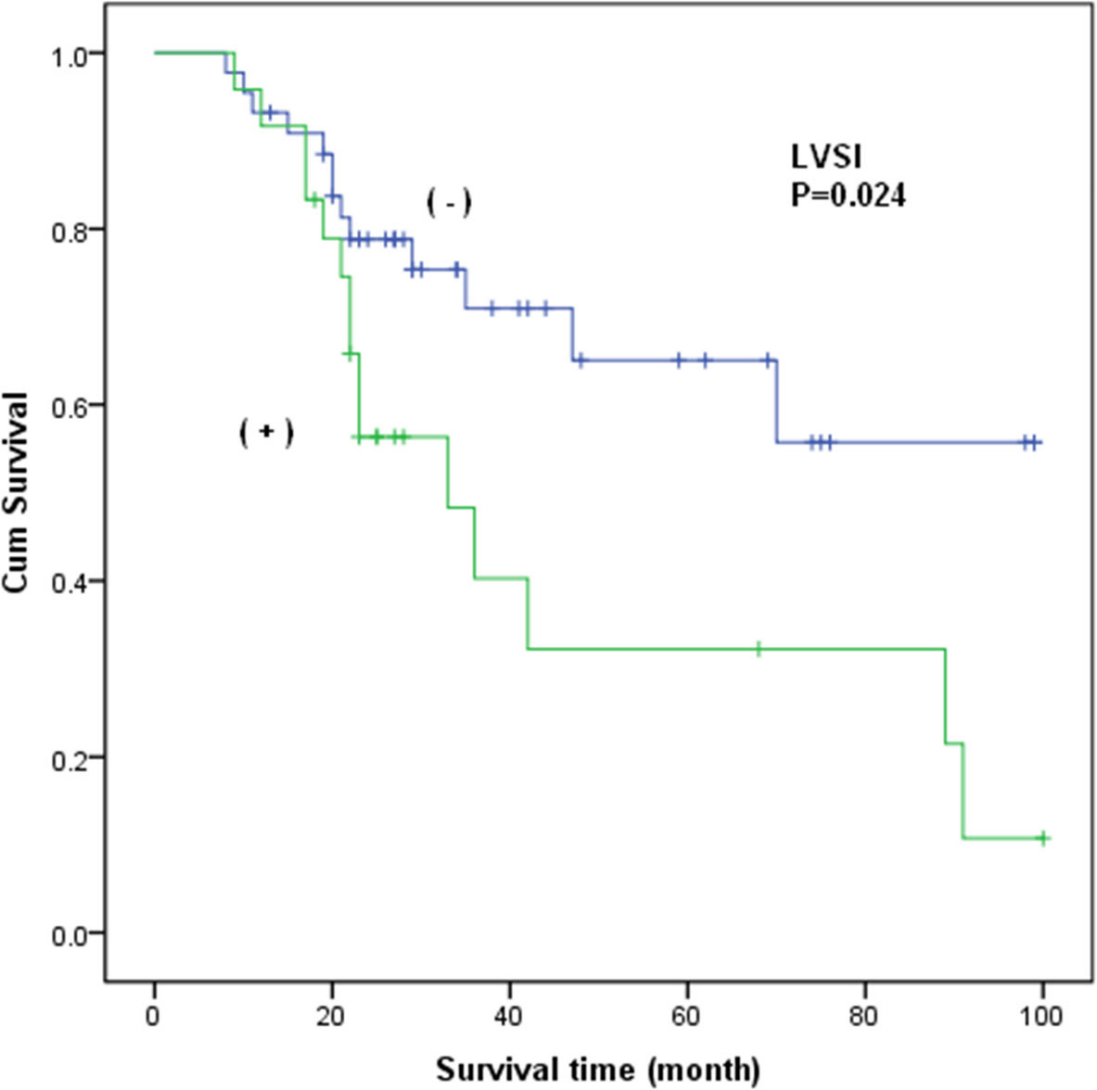
Kaplan-Meiercurves of overall survival for stage I-IIA patients with NECC by LVSI

To further evaluate the associations, data for a total of 76 stage I–IIA patients were retrieved. 68 patients underwent surgery as their primary treatment. The median overall survival of the patients was 60 months, and the estimated 3-year and 5-year overall survival rates were 65.4 and 47.7% respectively. As shown in Table 2, in the univariate analysis, the median survival time of patients with LNM was 45 months compared to 70 months for patients without LNM (HR = 2.723, 95%CI = 1.294–5.729; *P* = 0.008) (Fig. 2). Patients with LVSI exhibited a decreased median survival time (46 months) compared with patients without LVSI (69 months) (HR = 2.367, 95%CI = 1.121–4.999; *P* = 0.024) (Fig. 3). No association was found in the analyses of treatment modalities including NACT (*P* = 0.785) and RT (*P* = 0.129) with the survival of stage I–IIA patients with NECC.

**Table 2:** Stage I-IIA patients’ characteristics and clinical features

Parameters	Group (no.)	MST (mo)	HR	*P*
FIGO	I (53)	69		
	II (23)	49	2.291 (1.155–4.544)	0.018
Size	⩽4 cm (44)	67		
	>4 cm (32)	52	1.981 (0.997–3.937)	0.051
LNM	No (46)	70		
	Yes(22)	45	2.723 (1.294–5.729)	0.008
LVSI	No (44)	69		
	Yes (24)	46	2.367 (1.121–4.999)	0.024
DOI	⩽2/3 (53)	66		
	>2/3 (15)	32	2.102 (0.856–5.163)	0.105
NACT	No (34)	63		
	Yes (34)	61	1.111 (0.520–2.373)	0.785
Radiotherapy	No (49)	61		
	Yes (19)	37	1.864 (0.835–4.163)	0.129

The effect of RT on overall survival of stage I–IIA patients was investigated by stratified analysis of their clinicopathological features (Table 3). Among patients without LNM, those who did not receive RT had significantly better survival rate than those who received RT (HR = 3.363, 95%CI = 1.245–10.619; *P* = 0.018). In addition, among patients with DOI > 2/3, there seemed to be a poor survival for patients who received RT (HR = 2.338, 95%CI = 0.906–6.035; *P* = 0.079), although not statistically significant. Interestingly, the univariate analysis showed that patients with a tumor size > 4 cm who received NACT did not have a favourable survival than who did not receive NACT (*P* = 0.600). According to the multivariate analysis, none of the clinicopathologic features or treatment modalities was demonstrated to be a significant independent prognostic factor.

**Table 3:** Stratified analysis of treatment modalities associated with stage I-IIA patients’ survival

Parametres	RT (−)		RT (+)		HR	*P*
	No.	MST(mo)	No.	MST(mo)		
LNM (−)	34	80	12	39	3.363(1.245–10.619)	0.018
LNM (+)	15	43	7	47	0.581(0.157–2.157)	0.571
DOI (≤2/3)	9	25	6	35	0.486(0.087–2.706)	0.410
DOI (> 2/3)	40	71	13	43	2.338(0.906–6.035)	0.079
≤4 cm	28	76	10	39	0.371(0.115–1.195)	0.097
> 4 cm	21	59	9	44	1.363(0.464–4.139)	0.560
LVSI(−)	34	75	10	46	1.727(0.524–5.699)	0.369
LVSI(+)	15	51	9	36	1.385 (0.464–4.139)	0.560
	NACT(−)		NACT(+)			
> 4 cm	12	46	18	60	0.753(0.261–2.174)	0.600

## Discussion

Currently, due to the low incidence of NECC, most studies on NECC are comprised of limited series and case reports, which often do not include large sample sizes and prospective international studies. Therefore, it is difficult to establish appropriate guidelines for patients with NECC, only a small proportion of patients benefit from the current treatment options [6]. In our study, we performed a retrospective cohort study that consisted of 89 Chinese patients with NECC to investigate the potential prognostic factors and the optimal local treatment modalities for NECC.

The majority of stage I–IV patients (82.02%) in this study were treated with surgery as the primary treatment. The univariate analyses showed that features such as advanced FIGO stage, tumor size > 4 cm, LNM and LVSI exhibited a poorer survival, which suggests that they are high-risk factors for the recurrence and metastasis of NECC. However, the multivariate analyses showed that FIGO stage, tumor size, LNM and LVSI were not independent prognostic factors. The positive rate of LNM and LVSI in our study was 31.5 and 37%, respectively. However, a previous study of 188 patients showed that the positive rate of LNM and LVSI was 49.5 and 69.4%, respectively [1].

The 5-year OS rate of our patients with stage I-IIA NECC was 47.7%, which is consistent with the 5-year OS rates of 46.4, 46.6 and 45% for patients with stage I-II SCCC reported in previous studies [7–9]. In our study, radiation was given to 19 (27.9%) stage I–IIA patients. NACT was given to 34 out of the 68 stage I–IIA patients who underwent primary surgery. We have shown that neither RT (*P* = 0.129) nor NACT (*P* = 0.785) was prognostic factors for the survival of stage I–IIA patients with NECC. It has been demonstrated that those who received RT vs no RT had a 5-year survival of 6.4% vs 26.9%, respectively (*P =* 0.015), which suggests that RT resulted in no benefits on survival [1]. Another study showed that adjuvant chemoradiation did not improve survival compared with adjuvant chemotherapy alone [10]. The prognostic effects of RT and NACT according to pathological features were also examined among patients with stage I-IIA NECC. In contrary to our expectation, the patients without LNM who underwent surgery and who received postoperative RT did not have a significantly better survival rate than patients who did not receive RT. In agreement with our findings, a study by Huang et al. [11] found that patients who received RT tended to have a poorer survival rate than those who did not receive RT. Another study also reported that patients who received primary cancer-directed surgery (CDS) had a better survival rate compared with patients who received combined CDS and RT treatment or RT alone [8]. In addition, patients with LVSI, a tumor size > 4 cm or a DOI > 2/3, who received RT did not show any difference in overall survival, compared with those who did not receive RT. It indicated that RT might have little effect on disease control. The mechanism of poor survival of patients with no LNM who received postoperative RT remains to be elucidated. Perhaps the main reason is that those patients may have high-risk factors for metastasis and recurrence such as parametrial invasion and positive resection margins. Previous studies recommended NACT as the treatment of choice in patients with NECC with a tumor size > 4 cm [10, 12]. In contrast, Lee et al. [8] reported that patients who received NACT showed a poorer prognosis than those who did not receive NACT. Also, we found that patients who received NACT did not have a significantly lower risk of death compared with those who did not receive NACT. Based on these results, we suspect that NACT does not contribute to the reduced risk of metastasis and recurrence of NECC, although NACT might be useful for enhancing the resectability of bulky tumors. Moreover, patients who receive NACT usually have larger tumors, which indicate that the size of the tumor may affect patient survival. In addition, the development and prognosis of cancer is a complex and multifactor process. Genetic variants, in particular single nucleotide polymorphisms (SNPs), are associated with cancer survival. Next, we will assess the possible prognostic ability of genetic variants on NECC survival in future studies.

This study has some limitations that should be considered. First, due to the relatively small sample size of our study, other studies with larger sample size are needed to confirm our findings. Second, our study is based on a Chinese population; thus, the impact of each treatment modality on the survival outcome in different populations with NECC needs to be tested further. Third, chemotherapy is likely to enhance the survival of patients with stage IIB-IV NECC. However, we did not investigate the effects of chemotherapy because we did not have sufficient patient clinical information.

## Conclusion

In conclusion, advanced FIGO stage, tumor size > 4 cm, LNM and LVSI were associated with a significantly worse survival for patients with NECC according to the univariate analysis, but they were not independent prognostic factors in the multivariate analysis. Neither RT nor NACT showed a favourable effect among these patients. Furthermore, for stage I–IIA NECC, RT should be carefully used in patients without LNM. NACT may not be the optimal treatment for tumor size > 4 cm.

## Abbreviations

CDS: Cancer directed surgery
CgA: Chromogranin
DOI: Depth of stromal invasion
EP: Etoposide and cisplatin
GCIG: The Gynecologic Cancer InterGroup
LNM: Lymph nodemetastasis
LVSI: And lymph–vascular space invasion
MST: The median survival time
NACT: Neoadjuvant chemotherapy
NECC: Neuroendocrine cervical carcinoma
PACT: Postoperative adjuvant chemotherapy
RT: Radiotherapy
SCCC: Small cell carcinoma of the cervix
SGO: Society of Gynecologic Oncology
SNPs: Single nucleotide polymorphisms
Syn: Synaptophysin

## References

[1] Cohen JG, Kapp DS, Shin JY, Urban R, Sherman AE, Chen LM (2010). Small cell carcinoma of the cervix: treatment and survival outcomes of 188 patients. Am J Obstet Gynecol.

[2] Gardner GJ, Reidy-Lagunes D, Gehrig PA (2011). Neuroendocrine tumors of the gynecologic tract: a society of gynecologic oncology (sgo) clinical document. Gynecol Oncol.

[3] Cohen JG, Chan JK, Kapp DS (2012). The management of small-cell carcinomas of the gynecologic tract. CurrOpinOncol.

[4] EMcCusker M, RCoté T, XClegg L, JTavassoli F. Endocrine tumors of the uterine cervix: incidence, demographics, and survival with comparison to squamous cell carcinoma. Gynecol Oncol. 2003;8(3):333–9. 10.1016/s0090-8258(02)00150-6 12648583

[5] Landoni F, Maneo A, Colombo A, Placa F, Milani R, Perego P (1997). Randomised study of radical surgery versus radiotherapy for stage ib-iia cervical cancer. Lancet (London, England).

[6] Chan JK, Loizzi V, Burger RA (2003). Prognostic factors in neuroendocrine small cell cervical carcinoma: a multivariate analysis. Cancer.

[7] Da YL, Chong C, Lee M (2016). Prognostic factors in neuroendocrine cervical carcinoma. Obstet Gynecol.

[8] Zhou J, Yang HY, Wu SG, He ZY, Lin HX, Sun JY (2016). The local treatment modalities in figo stage i-ii small-cell carcinoma of the cervix are determined by disease stage and lymph node status. Cancer Med.

[9] Chin K, Baba S, Hosaka H, Ishiyama A, Mizunuma N, Shinozaki E (2008). Irinotecan plus cisplatin for therapy of small-cell carcinoma of the esophagus: report of 12 cases from single institution experience. Jpn J Clin Oncol.

[10] Lee JM, Lee KB, Nam JH, Ryu SY, Bae DS, Park JT (2008). Prognostic factors in figo stage ib–iia small cell neuroendocrine carcinoma of the uterine cervix treated surgically: results of a multi-center retrospective korean study. Ann Oncol.

[11] Huang L, Liao LM, Liu AW, Wu JB, Cheng XL, Lin JX, et al. Analysis of the impact of platinum-based combination chemotherapy in small cell cervical carcinoma: a multicenter retrospective study in chinese patients. BMC Cancer. 2014;14(1):1–9. 10.1186/1471-2407-14-140 PMC3939817 24575810

[12] Noda K, Nishiwaki Y, Kawahara M, Negoro S, Sugiura T, Yokoyama A (2002). Irinotecan plus cisplatin compared with etoposide plus cisplatin for extensive small-cell lung cancer. New Engl J Med.

